# Draft genome sequence of *Halomonas sulfidivorans* SD01 isolated from salt farm

**DOI:** 10.1128/mra.00568-25

**Published:** 2026-01-12

**Authors:** Sonam Dubey, Tejas C. Bosamia, Vijay Anand K. Gopalakrishnan, Arup Ghosh

**Affiliations:** 1 Applied Phycology and Biotechnology, CSIR—Central Salt and Marine Chemicals Research Institute 29016 , Bhavnagar, Gujarat, India; 2 CSIR—Central Salt and Marine Chemicals Research Institute 29016 , Bhavnagar, Gujarat, India; University of Southern California, Los Angeles, California, USA

**Keywords:** draft genome, polyhydroxyalkanoate, genes

## Abstract

The draft genome sequence of *Halomonas sulfidivorans*, a halophilic bacterium from the class Gammaproteobacteria, isolated from the experimental salt farm of CSIR—CSMCRI, Bhavnagar. Genomic analysis reveals the presence of genes involved in the synthesis of polyhydroxybutyrate.

## ANNOUNCEMENT

*Halomonas sulfidivorans* (SD01) was isolated from an experimental salt farm in Kumbharvada, Bhavnagar, Gujarat, India (21°47′26.4″N, 72°07′19.2″E) and cultured at 35°C, 120 rpm, pH 7. The isolation methods, media, sampling, and sample treatment are described by Dubey and Mishra (1). The strain was grown overnight in Zobel Marine broth supplemented with 3% NaCl for sequencing.

The taxonomic identity of the isolate was confirmed by 16S rRNA gene sequencing. Full-length 16S rRNA was amplified using colony PCR with universal primers 27F (5′-AGAGTTTGATCMTGGCTCAG-3′) and 1492R (5′-TACGGYTACCTTGTTACGACTT-3′), and sequencing was performed by Eurofins Genomics (Bangalore, India). The sequence was deposited in NCBI (accession no. PV242261.1) and showed 96.66% identity with *H. sulfidivorans* MCCC (accession no. CP053383.1).

For whole-genome sequencing (WGS), the same culture conditions were used. High-quality genomic DNA was extracted using the QIAamp DNA Mini Kit (Qiagen, Germany). Libraries were prepared with the QIAseq FX DNA Library Kit (Qiagen, Germany) following the manufacturer’s protocol. WGS was performed by Eurofins Genomics, covering all steps from DNA extraction to sequencing, using a whole-genome shotgun strategy on the Illumina NovaSeq 6000 platform (2 × 150 bp paired-end chemistry). Sequencing generated 8,421,488 reads (~2.4 Gbp), which were quality-processed with fastp v0.24.0, yielding 7,999,132 high-quality reads and final genome coverage of 530×.

Genome assembly was carried out using Shovill v1.1.0 (2), integrating the SPAdes assembler. To improve and polish the assembly, Pilon v1.2.0 (3) was employed for error correction, and further refinement was performed using RagTag v2.1.0 (4) for scaffolding, with *H. sulfidivorans* as the reference genome. RagTag does not alter the input query sequence but only reorders and orients contigs based on the reference genome. Default parameters were used for all bioinformatics tools used in the present study. The final draft genome assembly consisted of 98 scaffolds comprising 98 contigs, with a total length of 4,330,213 bp, an N50 for both contigs and scaffolds of 198.8 kb, and a G + C content of 64%. Genome assembly completeness was assessed using BUSCO v5, based on the Gammaproteobacteria ortholog set, revealing 99.4% completeness. Genome annotation was performed using the PGAP pipeline (5). The genome comprises 4,120 total genes, which include 4,001 protein-coding genes, 62 tRNAs, 11 rRNAs, 4 non-coding RNAs, and 42 pseudogenes. To determine the taxonomic identity of the isolate, fastANI v1.3 (6) was used to calculate the average nucleotide identity (ANI) between our isolate and the genomes of these five closely related *Halomonas* and *Billgrantia* species. The analysis revealed that our isolate shared the highest ANI (97.26%) with *H. sulfidivorans* strain MCCC (GenBank accession GCF_017868935.1), confirming that it represents a novel strain of *H. sulfidivorans*. The genes acetyl-CoA acetyltransferase (phaA), acetoacetyl-CoA reductase (phaB), polyhydroxyalkanoate (PHA) synthase (phaC), phasin family protein (phasin), and PHA synthesis repressor (phaR), all involved in the putative PHA biosynthesis pathway, are present in the genome sequence.

## Data Availability

This whole-genome shotgun project has been deposited at GenBank under accession number GCF_048575555.1. The raw sequencing reads are available at the Sequence Read Archive under accession number SRR32651082 and are associated with BioProject accession number PRJNA1233882.

## References

[1] Dubey S, Mishra S. 2021. Efficient production of polyhydroxyalkanoate through halophilic bacteria utilizing algal biodiesel waste residue. Front Bioeng Biotechnol 9:624859. doi:10.3389/fbioe.2021.624859 34604181 PMC8481892

[2] Seemann T. 2017. Shovill: Faster SPAdes assembly of Illumina reads

[3] Walker BJ, Abeel T, Shea T, Priest M, Abouelliel A, Sakthikumar S, Cuomo CA, Zeng Q, Wortman J, Young SK, Earl AM. 2014. Pilon: an integrated tool for comprehensive microbial variant detection and genome assembly improvement. PLoS One 9:e112963. doi:10.1371/journal.pone.0112963 25409509 PMC4237348

[4] Alonge M, Soyk S, Ramakrishnan S, Wang X, Goodwin S, Sedlazeck FJ, Lippman ZB, Schatz MC. 2019. RaGOO: fast and accurate reference-guided scaffolding of draft genomes. Genome Biol 20:224. doi:10.1186/s13059-019-1829-6 31661016 PMC6816165

[5] Li W, O’Neill KR, Haft DH, DiCuccio M, Chetvernin V, Badretdin A, Coulouris G, Chitsaz F, Derbyshire MK, Durkin AS, Gonzales NR, Gwadz M, Lanczycki CJ, Song JS, Thanki N, Wang J, Yamashita RA, Yang M, Zheng C, Marchler-Bauer A, Thibaud-Nissen F. 2021. RefSeq: expanding the Prokaryotic Genome Annotation Pipeline reach with protein family model curation. Nucleic Acids Res 49:D1020–D1028. doi:10.1093/nar/gkaa1105 33270901 PMC7779008

[6] Jain C, Rodriguez-R LM, Phillippy AM, Konstantinidis KT, Aluru S. 2018. High throughput ANI analysis of 90K prokaryotic genomes reveals clear species boundaries. Nat Commun 9:5114. doi:10.1038/s41467-018-07641-9 30504855 PMC6269478

